# Aging and chronic inflammation: highlights from a multidisciplinary workshop

**DOI:** 10.1186/s12979-023-00352-w

**Published:** 2023-06-08

**Authors:** Danay Saavedra, Ana Laura Añé-Kourí, Nir Barzilai, Calogero Caruso, Kyung-Hyun Cho, Luigi Fontana, Claudio Franceschi, Daniela Frasca, Nuris Ledón, Laura J. Niedernhofer, Karla Pereira, Paul D. Robbins, Alexa Silva, Gisela M. Suarez, Wim Vanden Berghe, Thomas von Zglinicki, Graham Pawelec, Agustín Lage

**Affiliations:** 1 Department of Clinical Immunology, Center of Molecular Immunology, 216 St, Corner 15, PO Box 16040, Atabey, Havana Cuba; 2 Albert Einstein College of Medicine, Bronx, United States; 3 Laboratorio di Immunopatologia e Immunosenescenza, Dipartimento di Biomedicina, Neuroscienze e Diagnostica Avanzata, Università di Palermo, Palermo, Italy; 4 LipoLab, Yeungnam University, Gyeongsan, Republic of Korea; 5 Raydel Research Institute, Medical Innovation Complex, Seoul, Republic of Korea; 6 Charles Perkins Centre, The University of Sydney, Sydney, Australia; 7 Institute of Biology and Biomedicine, Lobachevsky State University of Nizhny Novgorod, Nizhny Novgorod, Russian Federation; 8 Department of Microbiology and Immunology, University of Miami Miller School of Medicine, Miami, FL USA; 9 University of Minnesota Medical School, Minneapolis, MN USA; 10 Laboratory of Protein Chemistry, Proteomics and Epigenetic Signalling (PPES), University of Antwerp, Wilrijk, 2610 Belgium; 11 Integrated Personalized & Precision Oncology Network (IPPON), University of Antwerp, Wilrijk, 2610 Belgium; 12 Department of Biomedical Sciences, University of Antwerp, Wilrijk, 2610 Belgium; 13 Ageing Biology Laboratories, Newcastle University Biosciences Institute, Newcastle upon Tyne, UK; 14 Department of Immunology, University of Tübingen, Tübingen, Germany

**Keywords:** Aging, Chronic inflammation, Immunosenescence, Inflammaging, Cell senescence, SASP, Senolytic, Metformin, Biomodulina T

## Abstract

Aging is a gradual, continuous series of natural changes in biological, physiological, immunological, environmental, psychological, behavioral, and social processes. Aging entails changes in the immune system characterized by a decrease in thymic output of naïve lymphocytes, an accumulated chronic antigenic stress notably caused by chronic infections such as cytomegalovirus (CMV), and immune cell senescence with acquisition of an inflammatory senescence-associated secretory phenotype (SASP). For this reason, and due to the SASP originating from other tissues, aging is commonly accompanied by low-grade chronic inflammation, termed “inflammaging”. After decades of accumulating evidence regarding age-related processes and chronic inflammation, the domain now appears mature enough to allow an integrative reinterpretation of old data. Here, we provide an overview of the topics discussed in a recent workshop “Aging and Chronic Inflammation” to which many of the major players in the field contributed. We highlight advances in systematic measurement and interpretation of biological markers of aging, as well as their implications for human health and longevity and the interventions that can be envisaged to maintain or improve immune function in older people.

## Introduction

Aging is associated with changes in biological, physiological, immunological, environmental, psychological, behavioral, and social processes [1]. These changes are represented by hallmarks of aging that include genomic instability, telomere attrition, epigenetic alterations, loss of proteostasis, deregulated nutrient-sensing, mitochondrial dysfunction, cellular senescence, stem cell exhaustion, and altered intercellular communication. However, the aging process is nonlinear. A key word to characterize it could be “heterogeneity” as the aging/lifespan quality trajectories diverge largely among individuals [2]. This process is highly context-dependent. Everyone ages differently because each individual is unique in terms of genetics and life history, termed the “exposome”, which is the measure of all the exposures of an individual in a lifetime. Immunologically, the combination of type, intensity, and temporal sequence of antigens we are exposed to over the whole lifespan are of extreme importance in determining the “immunobiography” of the individual. This uniqueness can explain how the same antigenic molecule, depending on the genetics and the immunobiography of the host, can become either a strong or a weak antigen or can induce tolerance [3].

Aging of an individual is well known to be related to functional changes of immunity, resulting from age-associated variations in both the innate and the adaptive branches of the immune system. These changes occurring in the immune system with age are often referred to as “immunosenescence”, but this term should be reserved for those that have known negative consequences for the host [4]. It is important to note that immunosenescence is different, but related to cellular senescence, which refers to the irreversible exit from the cell cycle due to aberrant or extensive replication or different types of damage and stress including genotoxic and oxidative damage and inflammatory stress. Importantly, senescent cells acquire an inflammatory senescence-associated secretory phenotype (SASP). The architecture of the various lymphoid organs and structures where immune cells mature and are activated by antigen, exhibits marked aged-associated changes, as do the proportions of different immune cell subsets and their function [2]. Aging is also commonly accompanied by low-grade chronic inflammation, termed “inflammaging”. This low grade inflammatory process could represent a cost of maintaining immune surveillance against persistent pathogens or endogenous stressors throughout life [5, 6], or could be a solely pathological consequence of the aging process [7]. Recently, it has been suggested that inflammaging is mainly related to senescent cells and their associated SASP, which increase in the body with age and contribute to inflammaging and organismal aging [5, 6, 8]. One intriguing model even stated that aged immune cells are actually responsible for organismal aging [9].

After decades of accumulating phenomenology, frequently only loosely connected with the processes of aging, immunosenescence, cellular replicative senescence and chronic inflammation, the field seems now ripe to produce an integrative reinterpretation of old data, which could translate into clinically useful biological markers, identification of sensitive molecular targets and tools for interventions. The components of the processes (thymus involution, shrinkage of the immune repertoire, accumulation of memory and terminally differentiated lymphocytes, pro-inflammatory cytokines, senescent and SASP-secretory non-lymphoid cells, telomere shortening, overexpression of the cell cycle dependent kinase inhibitory genes p21^Cip1^ and p16^INK4a^, mitochondrial dysfunction, among many others) have been measured in different combinations and different settings, but there is still little insight as to the relationships of these phenomena to each other, which is required for discriminating what is real, what is relevant and what is actionable.

This was the focus of the hybrid workshop “Ageing and Chronic Inflammation”, organized as part of the BIOHABANA 2022 Meeting in April 2022, in Havana, Cuba. Academics and key opinion leaders in the field, immunologists, biologists, biochemists and geriatricians presented and discussed ideas about the associations among these processes, their implications for human health and longevity and the therapeutic approaches to target them. Some of the crucial points that emerged from the scientific presentations and associated discussions are reviewed below.

## Aging and immunosenescence

The concept of immunosenescence has evolved over the last two decades. The differences between younger and older adults observed in innate and adaptive immunity were first interpreted as being solely detrimental, assuming that the immune response is less efficient in eliminating pathogens, cancer cells, senescent cells and in responding to vaccines, leading to an increased susceptibility to infections and to the onset of age-related diseases [5]. However, a more nuanced view proposes a reinterpretation of many of the data with the knowledge resulting from more recent discoveries which suggest that not all changes in the immune system generally considered detrimental are actually harmful. In fact, age induces a decrease in immune functions but may also lead to increased function in certain aspects, which can be viewed as adaptive [5, 10]. Even in the case of responses to influenza vaccination, almost universally accepted as being greatly depressed in old people, the contribution of many factors including the nature of the vaccine and the biological rather than chronological age of the recipients can result in better responses of older relative to younger people [11].

An updated and more comprehensive concept of immunosenescence was recently put forward as a highly dynamic and multifactorial process, consisting of several changes in immune responses, where some functions decline sharply, while others are maintained or even increased, to varying degrees in the different subjects [2]. In most cases, data have accrued from cross-sectional rather than longitudinal studies, but here we will follow the majority of reports and refer to changes rather than differences, although it mostly remains an assumption that the differences measured do indeed represent changes with age, at least most of the time.

Changes in the adaptive branch of the immune system occurring with age have been the most intensively studied, and then mostly in mice and humans. Here, we focus on the latter. The most frequently described phenotypic differences between elderly and young individuals are: (i) the decrease in the naïve T cell populations, (ii) the increase in memory subpopulations principally in potentially terminally-differentiated T cells [2] which downregulate membrane expression of the CD28 receptor [12], likewise those which re-express the CD45RA marker [13]. These are mostly adaptive changes rather than necessarily maladaptive, even the decrease of naïve T cells with age, which is mostly a consequence of developmentally pre-programmed thymic involution and its direct impact on thymic function reduction [14]. The maintenance of a highly diverse and functional naïve T cell pool depends on the continuous replacement of peripheral naïve T cells. The age-dependent decline in naïve T cells in addition to the accumulation of memory T cell clones (due to life-long encounters with pathogens), leads to a reduction in the diversity of the T cell repertoire in elderly individuals, especially in CD4 + naïve T cells and in CD8 + late-differentiated memory cells [15]. This may result in a decreased capability to combat new pathogens as well as a decreased ability to mount vigorous recall responses for previously encountered pathogens, although robust data in support of this contention are limited in humans.

Additionally, the complex changes in acquired immunity are probably the result of epigenetic and metabolic modifications affecting immune cells. In younger people, the hematopoietic stem cells (HSCs) provide a balanced output of myeloid and lymphoid progenitor cells. An age-related shift from lymphoid to myeloid progenitors has been reported, suggesting the preferential differentiation of aged HSCs into common myeloid progenitor cells with the concomitant reduction in common lymphoid progenitor cell frequencies. This is followed by a reduction in T and B cell production with aging [16]. However, the reasons for this skewing of immune cell output from the bone marrow remain unclear.

There is much evidence indicating that chronic antigenic stimulation induced by the presence of persistent infections or by altered tissues and molecules, plays a major role in driving the peripheral T cell compartment into a state that is different in older individuals, possibly at least partially representing a state of exhaustion. A classic example of this process is latent cytomegalovirus (CMV) infection, which drives the accumulation of late-stage differentiated CD8 + T cells specific for this persistent herpesvirus [17]. As occurs with other herpesviruses, CMV establishes latency in the host and reactivates periodically especially under immunosuppressive conditions such as stress. The immunological consequence of keeping the virus in a latent state is the activation of a large proportion of CD4 + and CD8 + T cells against this virus, that precipitates an altered distribution of early- versus late-differentiated T cells in the periphery [18].

There has been a dearth of studies on populations other than those of the industrialized West, but comparative studies of other populations are beginning to emerge now, for example of Chinese [19] and Pakistanis [20]. A study in healthy Cubans showed that in a setting of high CMV seroprevalence (> 90%), the frequency of potentially terminally-differentiated and exhausted (PD1+) CD4 + and CD8 + T cells increases with age [21, 22]. Meanwhile, the naïve T cell compartment decreases (Saavedra D et al., manuscript in preparation). The Cuban population could be a particularly interesting cohort to study relationships between immunosenescence, inflammaging and chronic age-related diseases, due to the high antigenic load typical of a developing country in the tropical belt but coincident with low infant mortality, high life expectancy and an aged demographic pyramid, as a consequence of social interventions [22].

Although most of the literature on immunosenescence has focused on T cell changes, the B cell compartment is also different in older adults [23]. It is now clear that changes in B cells occur and have a significant impact on antibody production. The number of circulating B cells is reduced in the aged. Immunosenescence is also associated with a decrease in IgD + CD27- naïve cells and an increase of IgD-CD27- double negative late memory B cells which may also be exhausted memory cells [2]. Advanced age is also accompanied by specificity repertoire changes, modified peripheral B cell dynamics, and weakened humoral responses [24]. Notably, the human obese adipose tissue (AT), which increases in size with aging, contributes to systemic and B cell intrinsic inflammation reduced protective and increased pathogenic B cell responses leading to increased secretion of autoantibodies [25].

Two relevant issues in the current debate around the reinterpretation of immunosenescence are findings that the healthy elderly are able to sustain an adequate vaccine response compared with young subjects, and the increasing number of centenarians and semi-supercentenarians worldwide, mainly in the so-called blue zones [10].

As alluded to above, the most often cited vaccine failure in older adults is seasonal influenza, but while it is usually the case that the efficiency of this vaccine is lower in older than younger adults, this is not always true. The reasons for the differential responses are manifold. Frailty limits the ability of standard inactivated influenza vaccines to prevent hospitalization [26] and this is possibly due to a decline in T-cell responses, because antibody responses are relatively unaffected. In fact, surviving a prior influenza infection can restore influenza-specific T-cell responses on subsequent challenge by influenza vaccination. This suggests that poor immune stimulation reflects a limitation of current influenza vaccines rather than a limitation of the aging immune system [27]. Therefore, we need better vaccines, and there are many possibilities being investigated currently. A very recent vaccination success story is the unexpected efficacy of the COVID-19 vaccine in older adults [28]. Future vaccines should include changes in composition, adding of adjuvants, changes in doses, more mechanistic interventions such as the use of IL-7, among others. The challenge remains to identify the extrinsic (vaccine type) and intrinsic (frailty) factors predicting poor responsiveness at the individual level, in order to offer personalized protection not only against infectious disease but also possibly against cancer [29].

Centenarians are considered a model of successful aging because they succeed in preventing or delaying the onset of age-related diseases way beyond the average life expectancy. Centenarians may not actually avoid diseases but they are better able to resist their deleterious effects. The immune response of centenarians maintains an adequate functionality; it seems that they are able to control inflammaging [10]. A study in Cuban centenarians found that they had a good health status and were mainly only moderately dependent on others for their activities of daily living. They had low serum levels of IL-6, TNFα and C-reactive protein, and 40% of them had an inverted CD4/CD8 ratio which in some but not all studies represents a biomarker of incipient mortality [30]. Of note, despite 100% CMV seroprevalence, and the presence of immunosenescence markers and their association with frailty and survival beyond 100 years of age [31], there was no evidence of an association between inflammaging and survival in this cohort of Cuban centenarians (Ledon N et al., manuscript in preparation). This is a prime example of resistance to biological indicators that are detrimental to most of the population that does not reach such an advanced age. Data on Sicilian semi-super- and super-centenarians that show a slowdown in naïve T decline suggest that their maintenance of relatively healthy aging is linked to this slowdown, reinforcing the idea of the key role of this decline in the immunosenescence process [32].

Aging is by definition the single most important risk factor for all major age-related diseases and geriatric syndromes. However, the aging process is very different for each individual, which means that aging is far from uniform in every human being. Although there is an intense discussion regarding the use of specific biomarkers for the characterization of immunosenescence, and indeed, other biomarkers of aging, there is a consensus around the decrease in naïve cells and the increase in memory (senescent/exhausted) cells. Therefore, the decrease of naïve T and B cells, the accumulation of memory cells and the presence of inflammaging are considered “hallmarks” of immunosenescence [2] but in many cases their clinical relevance remains to be clarified.

## Inflammaging

As noted above, the term inflammaging indicates the low-grade chronic inflammatory status characteristic of the older individual. It was described for the first time as an explanation for the global reduction in efficient responses to new, as well as previously encountered antigens, concomitant with progressive increase in proinflammatory markers commonly seen in older individuals [33]. During the past decade, enough evidence has been collected indicating that different age-related diseases, such as atherosclerosis, cardiovascular diseases, type 2 diabetes, metabolic syndrome, osteoporosis, cognitive decline, neurodegenerative diseases and frailty have at least partially a common inflammatory pathogenesis [34, 35].

It has been stated that inflammaging and immunosenescence are two sides of the same coin. This means that there is a mutual interaction between the inflammaging-producing factors inducing immunosenescence and the immunosenescence-producing factors which contribute to the maintenance of the inflammaging [5]. The low-grade chronic inflammatory process described in older adults is characterized by increases in the levels of pro-inflammatory cytokines, such as IL-6. This pleiotropic cytokine has been associated with atherosclerosis, osteoporosis and sarcopenia, leading to functional decline, the development of disabilities and all-cause mortality [17]. Not only cytokines but also acute phase proteins, such as CRP and mannose-binding lectin, are markers of inflammaging [24].

Twenty years have now passed since the original introduction of the concept of inflammaging by Claudio Franceschi. During these years, it has been hypothesized that the inflammaging process is not developing exclusively from the cells of the innate or adaptive immune system. Inflammaging is also driven by (i) cell senescence (SASP); (ii) the imbalance of microbiome composition, in various parts of the organism, especially in the gut; (iii) the innate immune memory of trained innate immunity; and (iv) metabolic epigenetic changes induced by the mitochondria [3, 6, 36]. An imbalance between commensal microbes and invasive microbes may occur at advanced age. Such invasive microbes may induce the production of proinflammatory mediators and enhance inflammation [6, 37]. The trained innate immunity concept proposes that because of epigenetic and metabolic changes, the innate immune system is in a state of chronic activation. This might be beneficial for the next response, that could be more efficient than the previous one. However, trained immunity could also be counterproductive and result in a paralyzed state, when crossing a threshold of no-return [6, 38]. The metabolic changes manifested by the mitochondria during aging may also contribute to inflammaging. Mitochondria may increase the production of free radicals and the release of damage components into the cytosol that could be detected by the pattern recognition receptors, leading to innate inflammatory response [6, 39, 40]. Moreover, an important contribution to inflammaging may also derive from senescent cells [6, 34].

## Cell senescence and chronic inflammation

Cellular senescence is a cell fate characterized by irreversible cell-cycle arrest with secretory features, macromolecular damage, and altered metabolism. It is implicated in various physiological processes in addition to aging, and is associated with a wide spectrum of age-related diseases. Despite the name, therefore, cellular senescence is not a synonym for aging and is not exclusive to advanced age or pathologic processes. A cell can initiate the senescence program regardless of organismal age. It is present from the moment of embryogenesis, contributing to tissue development, and later on, in adulthood, plays a role in tissue repair and tumor suppression [41, 42]. Based on this duality of beneficial and detrimental effects, cellular senescence has been proposed to be an example of evolutionary antagonistic pleiotropy [43].

Senescence primarily associated with detrimental effects can be triggered by a number of stress signals to the cell, including DNA damage, telomere shortening or dysfunction, oncogene activation or loss of tumor suppressor functions, mitochondrial dysfunction, nutrient deprivation, hypoxia and epigenetic changes [44]. The main cause of senescent stress is DNA damage, which activates the DNA damage response (DDR) and the canonical p53–p21 pathway, and in consequence leads to cell-cycle arrest [42, 45]. The overexpression of p21^Cip1^ and p16^INK4A^ is characteristic of senescent cells (but not exclusively of senescent cells), and is widely recognized nowadays as a cellular senescence marker, especially together with telomere dysfunction. However, a single marker cannot be used to asses senescence. Instead, a comprehensive multi-marker approach including evaluation of other cellular senescence hallmarks is needed [41]. Other characteristic traits of senescent cells include increased SA-ßgal activity, larger morphology, altered nuclear structure, changes in heterochromatin and the high production of reactive oxygen species (ROS) due to impaired mitochondrial function, termed senescence-associated mitochondrial dysfunction (SAMD) [46]. SAMD is able to drive NF-kB activation in cell senescence, which induces the SASP.

The SASP constitutes a hallmark of senescence and mediates many of its patho-physiological effects. Senescent cells secrete bioactive molecules, especially pro-inflammatory cytokines and chemokines contributing to systemic sterile chronic inflammation associated with age-related diseases, frailty and mortality in the elderly. However, the SASP includes more than pro-inflammatory factors, since ROS, growth factors, matrix-remodelling factors, (non-coding) RNAs as well as other peptides and proteins can be part of the phenotype. Moreover, SASP composition and intensity varies depending on the pro-senescence stimulus, the duration of senescence, and cell type and microenvironment. So, the senescent secretome is different under different biological conditions [41].

As emphasized in the meeting, there is a close association between chronic inflammation and cell senescence. The SASP reinforces and spreads senescence in an autocrine and a paracrine manner [47–49]. This ability of senescent cells to induce a senescent phenotype in surrounding cells through the SASP has been termed bystander senescence. Thus, a positive feedback loop is established, in which senescence causes chronic inflammation and inflammation causes senescence [49].

Senescent cells accumulate with age in multiple tissues and may cause functional decline. In the immune system, senescence affects both innate and adaptive immunity, in particular follicular helper T cell and natural killer cell function. In order to define the contribution of immune system aging to organism aging a mouse model with a selective deletion of a DNA damage repair protein in hematopoietic cells was generated to induce senescence in the immune system only. Remarkably, non-lymphoid organs from these mice also exhibited increases in senescence markers, which suggests that a senescent immune system has a causal role in driving systemic aging [9].

## Possible therapeutic strategies

Because aging is the most significant risk factor for many diseases and conditions, targeting the aging process itself could have a large impact on human health. However, an increased understanding of aging phenomena and mechanisms must be followed by interventions aiming to improve human health. Different ways and means are being explored to improve immune function in older adults. These strategies include low-tech approaches such as programs of physical exercise and healthy nutrition. Many signs of immunosenescence could be exacerbated by decreased physical activity often seen in older adults. Consistent with this, the age-associated decrease of naïve T cells could be partially prevented in older adults who maintained high levels of physical activity throughout adult life [50]. More experimental trials have been performed on the restoration of a “young” thymic environment with agents such as interleukin-7 (IL-7), hormones, growth factors, thymic peptides [51] and the use of senolytic drugs that have all been proposed recently [2].

In this context, at BIOHABANA 2022, several proposals were discussed, which could be considered as of two types: non-pharmacological and pharmacological. Within the non-pharmacological interventions, several studies were presented showing the effects of consumption of polyphenols contained in cocoa and those related to dietary restriction without malnutrition. There are several lines of evidence about how the consumption of certain flavonoids in fruit, vegetables and cocoa can modulate important networks of genes in blood cells involved in functional processes and interactions with the vascular endothelium, such as response to oxidative stress, cell-cell adhesion, apoptotic and senescence processes, or cellular transport. Due to their multiomic modes of action, there are no individual genes/proteins involved in mediating the healthy properties of these bioactive compounds, but rather a multi-targeted mode of action that leads to an overall significant change in global gene expression profile related to modulation of immune response and reduction of inflammation [52–54]. Here, also the gut microbiota is sure to play an important role too [55–59] .

Dietary restriction without malnutrition is the gold standard for delaying aging and extending life and health in various species. A thought-provoking analysis of the effects of dietary restriction, intermittent fasting and exercise on the production of physiological, metabolic, and molecular changes shows that those factors are responsible for the prevention of multiple diseases associated with aging in humans. In particular, moderate dietary restriction in humans ameliorates multiple metabolic and hormonal factors that are implicated in the pathogenesis of type 2 diabetes, cardiovascular diseases, and cancer, the leading causes of morbidity, disability and mortality [60]. Furthermore, different forms of fasting and protein restriction, selectively impact the activity of AKT, FOXO, mTOR, NAD+, AMPK, and fibroblast growth factor 21 (FGF21), which are key components of some of the most important nutrient-sensing geroprotective signalling pathways that promote healthy longevity. A crucial point to consider is that experiments have demonstrated that genetic and epigenetic background determines the response to dietary interventions, including dietary restriction in mice. It is therefore very important that these findings will be clinically translated using a personalized food-as-medicine approach to identify how each person can improve his or her health and lifespan. This implies the need to educate the population on the benefits of a healthy diet and the limitations of the scientific consensus [61–63].

From the discussion in the sections above, it is clear that immune function is impaired with aging, leading to more severe infections and increased mortality. Several recent studies demonstrated that reducing the senescent cell burden and the inflammatory SASP by treatment with senolytic and senomorphic compounds improves the immune response and reduces mortality [64–67]. These observations have led to several clinical trials to test the effect of senolytics and senomorphics [68]. Interestingly, exposure to pathogens can increase the extent of senescence through both direct and indirect mechanisms, especially in older adults, driving further immune dysfunction, senescence and non-specific inflammation. This increase in inflammation driven by the SASP then contributes to increased mortality and morbidity [69, 70]. Importantly, these observations suggest that developing approaches to limit senescence in the adaptive and innate immune cells would not only improve the immune response, but might also slow aging [71].

Other drugs, such as metformin, may also modulate the hallmarks of aging by enhancing nutrient sensing, autophagy, intercellular communication and mitochondrial function, protecting against macromolecular damage, delaying stem cell aging and regulating transcription [72, 73]. These characteristics make metformin an attractive senomorphic and gerotherapeutic for anti-aging clinical trials, such as the TAME (Targeting Aging by MEtformin) clinical trial [74].

Two natural products from Cuba were presented at the workshop. Policosanol exerts its action through the improvement of the anti-inflammatory effect on high-density lipoproteins. It is a mixture of eight aliphatic primary alcohols purified from sugar cane wax (Saccharum officinarum L.). These primary alcohols range from 24 to 34 carbon atoms, with octacosanol, triacontanol, dotriacontanol, hexacosanol and tetratriacotanol as the main constituents. Policosanol improves the beneficial functions of HDL to maximize its antioxidant, antiglycation, and antiatherosclerotic activities, as well as cholesterol ester transfer protein inhibition. These improvements in HDL functionality could exert anti-aging and rejuvenating activity [75, 76].

The other agent, Biomodulina T (BT) is a polypeptide fraction obtained from the bovine thymus. The administration of BT to older adults with a history of recurrent respiratory infections resulted in an expansion of naïve CD4 + T cells, recent thymic emigrants and stem cell-like memory CD8 + T lymphocytes, whereas the proportions of CD4 + and CD8 + T cells expressing PD1 were decreased [77]. Putatively terminally-differentiated CD4 + EMRA and CD8 + EMRA T cells decreased after BT treatment, whereas CD4 + naïve T cells increased also in patients with advanced lung cancer, immediately after platinum-based chemotherapy. Additionally, CD4 + and CD8 + T cells expressing PD1 were also reduced after BT administration in cancer patients, highlighting its likely effects countering “exhaustion” [78]. Intervention with BT contributes to restoration of the normal thymic environment by slowing the reduction of the number of naïve T cells that occurs naturally during the aging process and may improve the efficacy of immunotherapy in older adults susceptible to recurrent infections and cancer [79].

## Concluding remarks

Healthy human lifespan has been rapidly extended during the XIX and XX centuries, historically to a large extent by decreasing early mortality. Any further expansion must occur in the post-reproductive life, where natural selection of adaptive genetic traits does not occur anymore, no biological mechanism can be expected to drive the process. Success will come from interventions into human aging, both social and biological, that address primarily healthspan, not only lifespan.

Progress towards interventions in human aging will be a complex task. Aging is multifactorial and therefore no single molecular measurement can be efficient to stratify the human population or to monitor the impact of interventions. We will need multivariate analysis of data, multicomponent indexes, and cluster identification, in order to move beyond chronological age measurement and to build a useful biological clock for human life. In a recent breakthrough, biomarkers of ageing based on DNA methylation data have enabled accurate age estimates for any tissue across the entire life course. These ‘epigenetic clocks’ link developmental and maintenance processes to biological ageing, giving rise to a unified theory of life course [80, 81]. Although it is already known for years that cumulative epigenetic changes occur upon aging, DNA methylation patterns were only recently used to construct an epigenetic clock predictor for biological age, which is a measure of how well your body functions compared to your chronological age. Today, this epigenetic DNA methylation clock signature is increasingly applied as a biomarker to estimate aging disease susceptibility and mortality risk. Moreover, the epigenetic clock signature could be used as a lifestyle management tool to monitor healthy aging, to evaluate preventive interventions against chronic aging disorders and to extend healthy lifespan [62]. Dissecting the mechanism of the epigenetic aging clock will yield valuable insights into the aging process and how it can be manipulated to improve health span [82, 83].

Clinical trial designs will be challenging as aging is not a disease, and several age-associated changes reflect successful adaptation and not malfunction, as illustrated by data in centenarians.

**Fig. 1:**
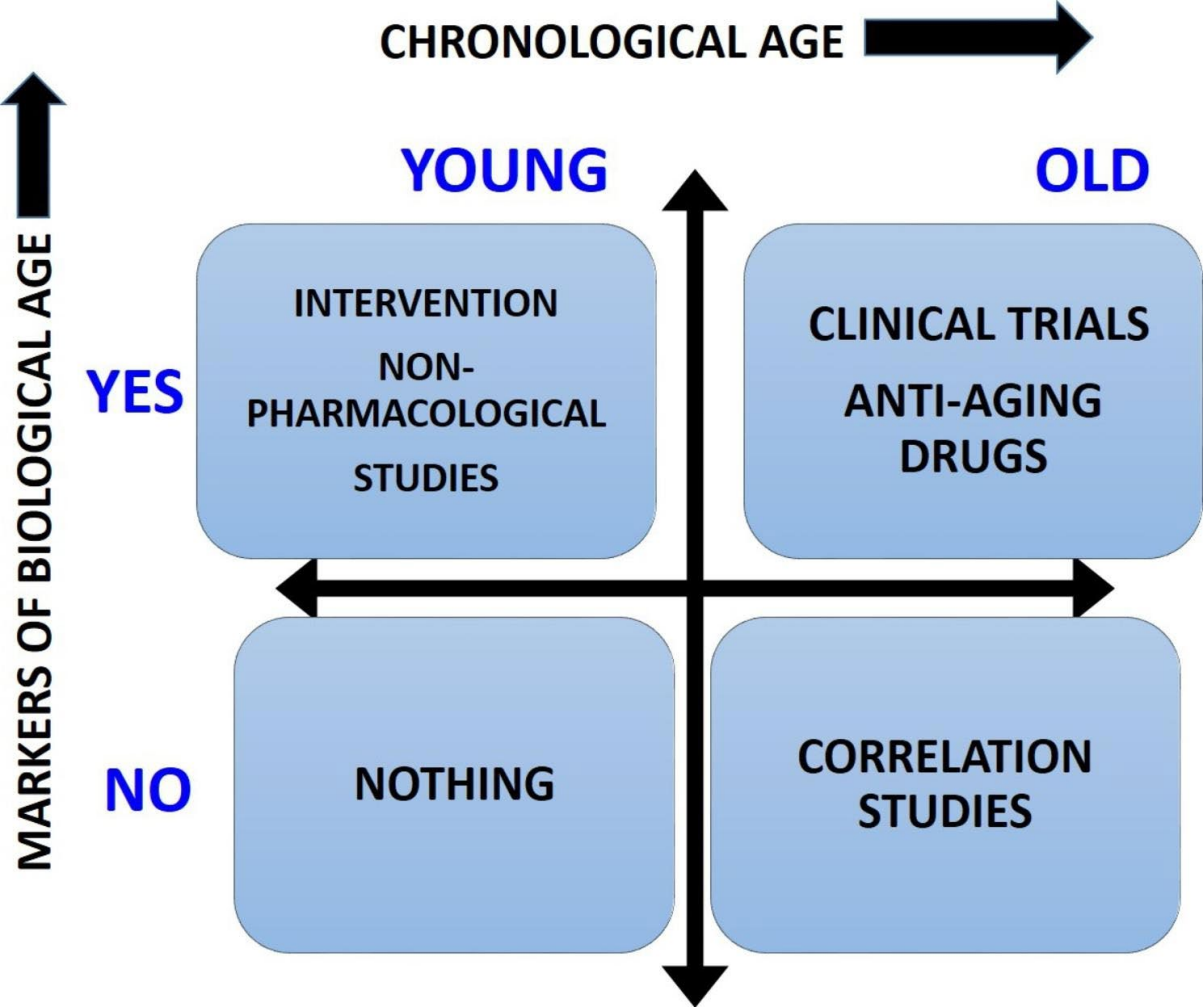
2 × 2 matrix representing biological markers of aging (biological age) and chronological age

Systematic measurements of biological markers of ageing (biological age), and their interpretation, together with chronological age, could define four groups of people, as depicted in the following 2 × 2 matrix (Fig. 1).

This double stratification could help to tailor intervention strategies according to both biological and chronological age simultaneously. Young people without inflammatory markers would not require specific interventions beyond general health counseling. Old people but without inflammatory markers deserve further observation and longitudinal follow up. Persons that are young but express markers of inflammaging or immunosenescence could be the subjects for trials of non-pharmacological interventions (nutrition, exercise, and life style), whereas old people showing markers of inflammaging or immunosenescence could be the eligible population for clinical trials of drugs.

To build and to validate a multivariate index, including measurements able to provide non-redundant predictive power for meaningful clinical events, to stratify the human population according to these clusters, to develop new products targeting not only specific molecular markers for specific age-related disease but also the underlying senescence processes, are the challenges to face before the next Workshop.

## Data Availability

Not applicable.
